# Aberrant expression of WDR4 affects the clinical significance of cancer immunity in pan-cancer

**DOI:** 10.18632/aging.203284

**Published:** 2021-07-19

**Authors:** Hanqian Zeng, Shiwen Xu, Erjie Xia, Suzita Hirachan, Adheesh Bhandari, Yanyan Shen

**Affiliations:** 1 Department of Breast Surgery, The Second Affiliated Hospital of Wenzhou Medical University, Wenzhou, Zhejiang Province, China; 2 Department of Breast Surgery, The First Affiliated Hospital of Wenzhou Medical University, Wenzhou, Zhejiang Province, China; 3 Department of Surgery, Breast Unit, Tribhuvan University Teaching Hospital, Kathmandu, Nepal

**Keywords:** pan-cancer, prognostic biomarker, WDR4, immune infiltration, clinical significance

## Abstract

Recent publications have presented research showing that WD repeat domain 4 (WDR4) plays a significant role in various kinds of malignant tumours. However, the expression profile of WDR4 is still unspecified, as is its significance in the analysis of human pan-cancer. We conducted an in-depth analysis of three aspects of WDR4 expression patterns from 33 types of cancer and determined the value of WDR4 for prognostic prediction and carcinoma drug resistance prediction. WDR4 was expressed in different cancer cell lines at inconsistent levels. Aberrant expression of WDR4 has been observed in various malignant cancers and is significantly implicated in overall survival outcomes. The expression level of WDR4 is also strongly associated with tumour immunity, such as immune scores and tumour-infiltrating immune cells. The level of WDR4 is related to microsatellite instability and tumour mutation burden in several types of malignancy, and validation studies implied that WDR4-associated terms and pathways are involved in malignancy. We explored the expression level of WDR4 across 33 types of cancer and showed that WDR4 plays a significant role during cancer development. More crucially, WDR4 is associated with immune infiltration, which suggests that WDR4 could be an immunotherapy target in cancers. In summary, our research showed that WDR4 plays a vital role in tumorigenesis and has the potential for to be targeted with treatments.

## INTRODUCTION

Over the recent years, the incidence of carcinomas has increased at a substantial rate worldwide, which is primarily attributable to lifestyle and improvements in healthcare methods for detecting tumours. Malignant tumours are one of the principal causes of mortality in both developing and developed nations, with limited therapeutic success achieved worldwide [1, 2]. Currently, pan-cancer investigations have been generally utilized to explore the common features or heterogeneities involved in the existence and development of cancer [3–5]. Pan-cancer analysis reveals the similarities and differences between the genomes and cell changes of numerous carcinoma types that can distinguish several mutual characteristics or heterogeneities in crucial biological processes [3]. Pan-cancer analysis resources, such as The Cancer Genome Atlas (TCGA), have been used for exome, transcriptome, and DNA methylome data analysis to draw a comprehensive picture of commonalities, differences, and emerging themes among tumour types [6–8]. Pan-cancer analysis has been applied to identify pathway genes, which permits the acquisition of a wide-range of in-depth knowledge of the molecular mechanisms linked to malignancy [9–11]. Ghoshdastider et al. inferred cross-talk among ligands and receptors on carcinoma and stromal cells in the TME of 20 types of solid tumour by analyzing tumour transcriptomes [12]. Distinctive characteristics of TIMs across carcinoma types were recently revealed in a pan-cancer investigation of single myeloid cells from a total of 210 patients with 15 human carcinoma types [13]. Luo et al. identified the overexpression of TRF1 and POT1 and the coamplification/deletion of TRF2-RAP1-TPP1 as dominant alteration incidents by performing a complete investigation of shelterin in 9125 cancer patients with 33 types of tumour using multiomics data from The Cancer Genome Atlas [14]. Therefore, pan-cancer examination can be beneficial for investigating the occurrence of various tumours and for developing individualized therapies for treatment techniques.

WD repeat domain 4 (WDR4) is a member of the WD-repeat protein family, which is associated with a variety of cellular developments, including cell cycle evolution, signal transduction, gene regulation, and apoptosis [15–17]. WDR4 can negatively regulate PML via ubiquitination to drive lung tumour development by fostering the development of an immune-suppressive and premetastatic tumour microenvironment, indicating the potential of immune-modulatory methods for treating lung carcinoma [18]. Lee cc found that Wuho/WDR4 insufficiency leads to an increase in γH2AX protein levels, heterochromatin relaxation, and DNA impairment of downstream sequences, which prevents cell proliferation and leads to apoptosis [19]. To understand the functions of WDR4 in different tumours, a comprehensive analysis is essential.

In our study, we aimed to investigate the expression of WDR4 and its prognostic significance in human tumours using data from the TCGA. The involvement of WDR4 in tumour infiltration, microsatellite instability (MSI), and tumour mutational burden (TMB) was analyzed in various types of cancer. Gene set enrichment analysis (GSEA) was performed to explore the principal mechanisms. The results of this study provide information regarding the role of WDR4 in tumours, reveal the relationship between WDR4 and tumour-immune interactions, and clarify the potential underlying mechanisms.

## MATERIALS AND METHODS

### Patient datasets and processes

The patient datasets and processes were taken from The Cancer Genome Atlas (TCGA), a cornerstone of cancer genomics projects. (https://tcga-data.nci.nih.gov/tcga/). Our research comprises more than 20,000 initial tumour samples and corresponding non-carcinoma samples for 33 types of carcinoma. The data from the Cancer Cell Line Encyclopedia (CCLE) project were downloaded from the website http://www.broadinstitute.org/ccle. For this study, only open-access data were used, which excluded the requirement of authorization from the Ethics Committee.

### Differential expression of WDR4 and screening of cancers related to survival

WDR4 gene expression data was obtained from the TCGA to compare the expression levels among the carcinomatous and adjacent normal samples. A univariate Cox model was applied to evaluate the association between patient survival outcomes and WDR4 expression levels. A *P*-value of 0.05 was considered statistically significant. Kaplan-Meier (KM) analysis was implemented to evaluate the association between overall survival (OS) outcomes among TCGA carcinoma patients and the WDR4 expression level by the log-rank test, and a survival-associated forest plot was generated.

### WDR4 and tumour immunity

The tumour immunity estimation resource (TIMER, https://cistrome.shinyapps.io/timer/) is a complete method for the systematic study of the immune infiltration level of several types of carcinoma [20]. In TIMER, a deconvolution statistical technique is utilized to determine level of tumour-infiltrating immune cells based on gene expression data [21]. Using the TIMER algorithm, we investigated the association between WDR4 levels and the infiltration levels of 6 different immune cell types (CD4+ T cells, CD8+ T cells, B cells, neutrophils, dendritic cells, and macrophages).

TMB represents the number of alterations in a specific cancer genome. Many studies have revealed the importance of TMB as a predictive biomarker for patient checkpoint inhibitor sensitivity [22]. We acquired the somatic mutation data of TCGA patients (https://tcga.xenahubs.net), analysed the TMB scores, and determined the association between TMB and WDR4 expression levels. MSI is considered an extensive polymorphism of the microsatellite sequences resulting from DNA polymerase slippage. Recently, it was hypothesized that cancer patients with elevated MSI benefit from immunotherapy, and MSI has been used as a marker of genetic uncertainty in malignancy. We analysed the MSI score of every patient and then performed a correlation analysis between WDR4 expression levels and the MSI score.

### Gene set enrichment analysis

Additional gene set enhancement investigation was similarly achieved utilizing GSEA (Gene Set Enrichment Analysis) software v2.2.1 (Gene Set Enrichment Analysis, http://www.broadinstitute.org/gsea/index.jsp). When the number of random sample arrangements was 100 and the significance threshold was *P* < 0.05, R software (http://r-project.org/) and Bioconductor (http://bioconductor.org/) were applied to visualize our results.

### Statistical methods

The Wilcoxon log-rank test was applied to determine the significance of the noticeably increased gene expression z-scores of carcinogenic tissues in comparison with those of adjacent normal tissues. The difference in WDR4 expression levels among different cancer types was analyzed with the Kruskal-Wallis test. Survival outcomes were investigated by the log-rank test, Cox proportional hazards regression model and KM curves. For the correlation analysis, Spearman's test was applied.

### Ethical approval

Ethical approval for this study was obtained from the Ethics Committee of the Second Affiliated Hospital of Wenzhou Medical University.

## RESULTS

### Pan-cancer expression landscape of WDR4

**Figure 1:**
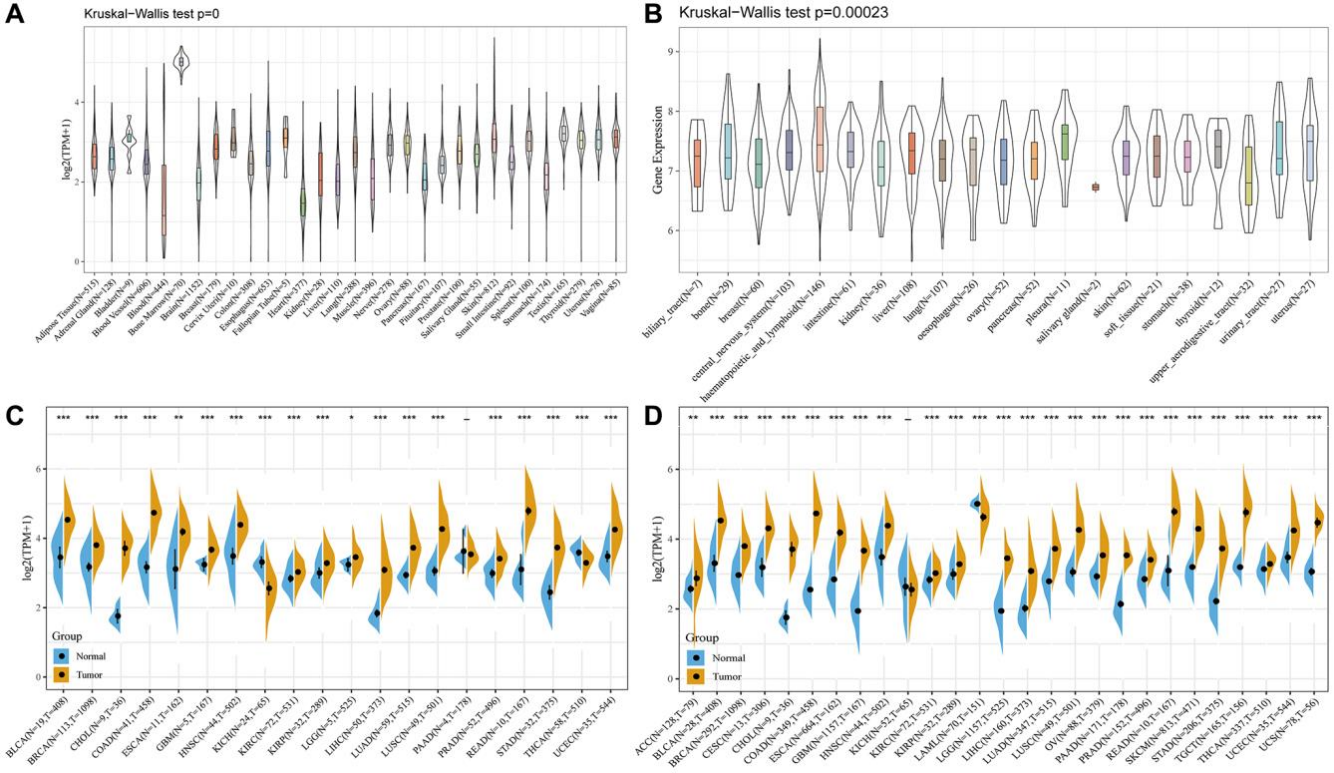
**The WDR4 expression level in human pan-cancer analyses.** (**A**) Expression of WDR4 in 31 tissues in GTEX. (**B**) Expression of WDR4 in 21 tissues in CCLE. (**C**) The level of WDR4 in TCGA. (**D**) The expression level in TCGA combined with GTEX. The blue and yellow bar graphs indicate normal and tumour tissues, respectively. ^*^P < .05; ^**^P < .01; ^***^P < .001.

The GTEX and CCLE analysis outcomes revealed that WDR4 gene expression levels were inconsistent across several cancer cell types (Figure 1A, 1B).

For most TCGA-derived malignancy types, we discovered significantly upregulated WDR4 expression between tumour samples and paired normal samples, except for in the TCGA-KICH and TCGA-PAAD cohorts (Figure 1C). Figure 1D shows that after combining the TCGA and GTEX analysis results, WDR4 expression was constantly upregulated in most cancer types, except for in the TCGA-KICH cohort.

**Figure 2:**
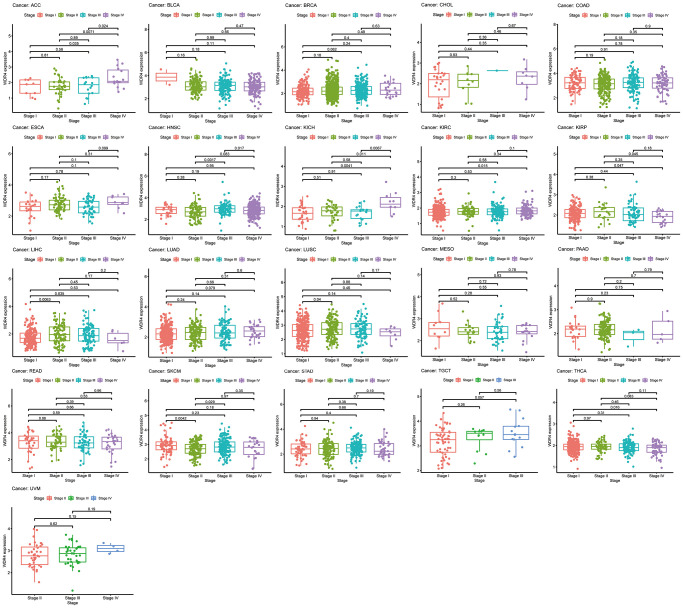
The box plot shows the association of WDR4 expression with pathological stages for 21 types of cancers.

To assess the WDR4 gene expression levels at different cancer stages, we measured WDR4 expression levels in patients with stage I, II, III, and IV disease. As illustrated in Figure 2, WDR4 expression was upregulated at the advanced stages in ACC, HNSC, KICH, KIRC, LUSC, LIHC, SKCM, and THCA, whereas it was downregulated in advanced KIRP tumours and was constant in advanced BLCA, BRCA, CHOL, COAD, ESCA, LUAD, MESO, PAAD, READ, STAD, TGCT, and UVM.

### Screening of the association between WDR4 expression and carcinoma survival outcomes

**Figure 3:**
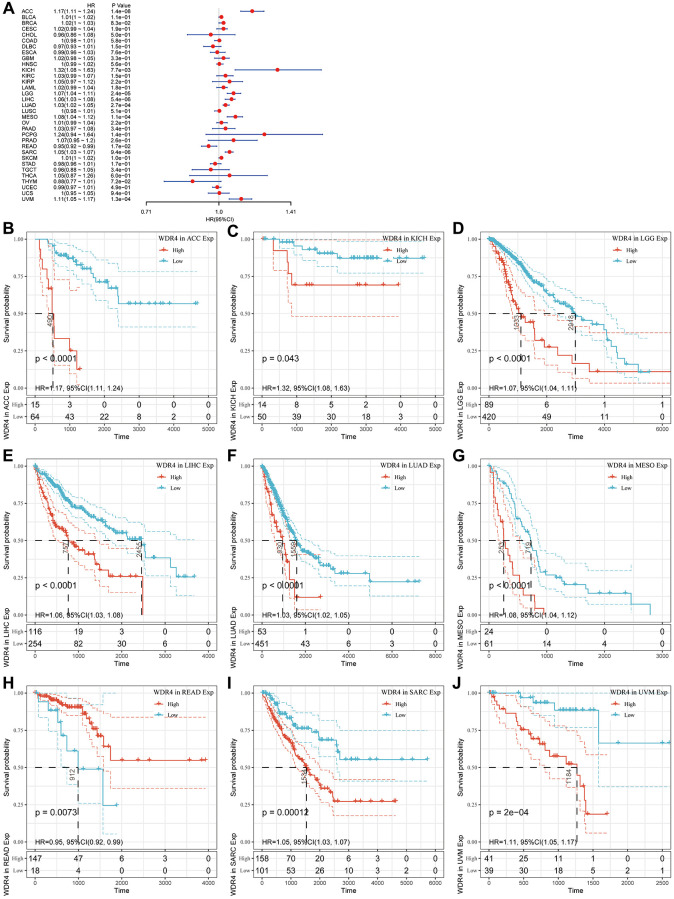
**Association of WDR4 expression with patient overall survival (OS).** (**A**) The forest plot shows the relationship of WDR4 expression with patient OS. (**B**–**J**) Kaplan-Meier analyses show the association between WDR4 expression and OS.

In the OS outcome study, Cox regression revealed that higher expression of WDR4 is a risk factor in ACC (*P* < .001), BLCA (*P* < .001), BRCA (*P* < .001), KICH (*P* < .001), LGG (*P* < .001), LIHC (*P* < .001), LUAD (*P* < .001), MESO (*P* < .001), SARC (*P* < .001), SKCM (*P* < .001) and UVM (*P* < .001); however, WDR4 expression appeared to be a protective factor in READ (*P* < .001) as shown in Figure 3A. KM analysis showed that patients with higher WDR4 levels had a shorter OS times compared with patients with low WRD4 levels in ACC (*P* < .001), KIRC (*P* = .043), LGG (*P* < .001), LIHC (*P* < .001), LUAD (*P* < .001), MESO (*P* < .001), READ (*P* < .001), SARC (*P* < .001) and UVM (*P* < .001), as illustrated in Figure 3B–3J.

**Figure 4:**
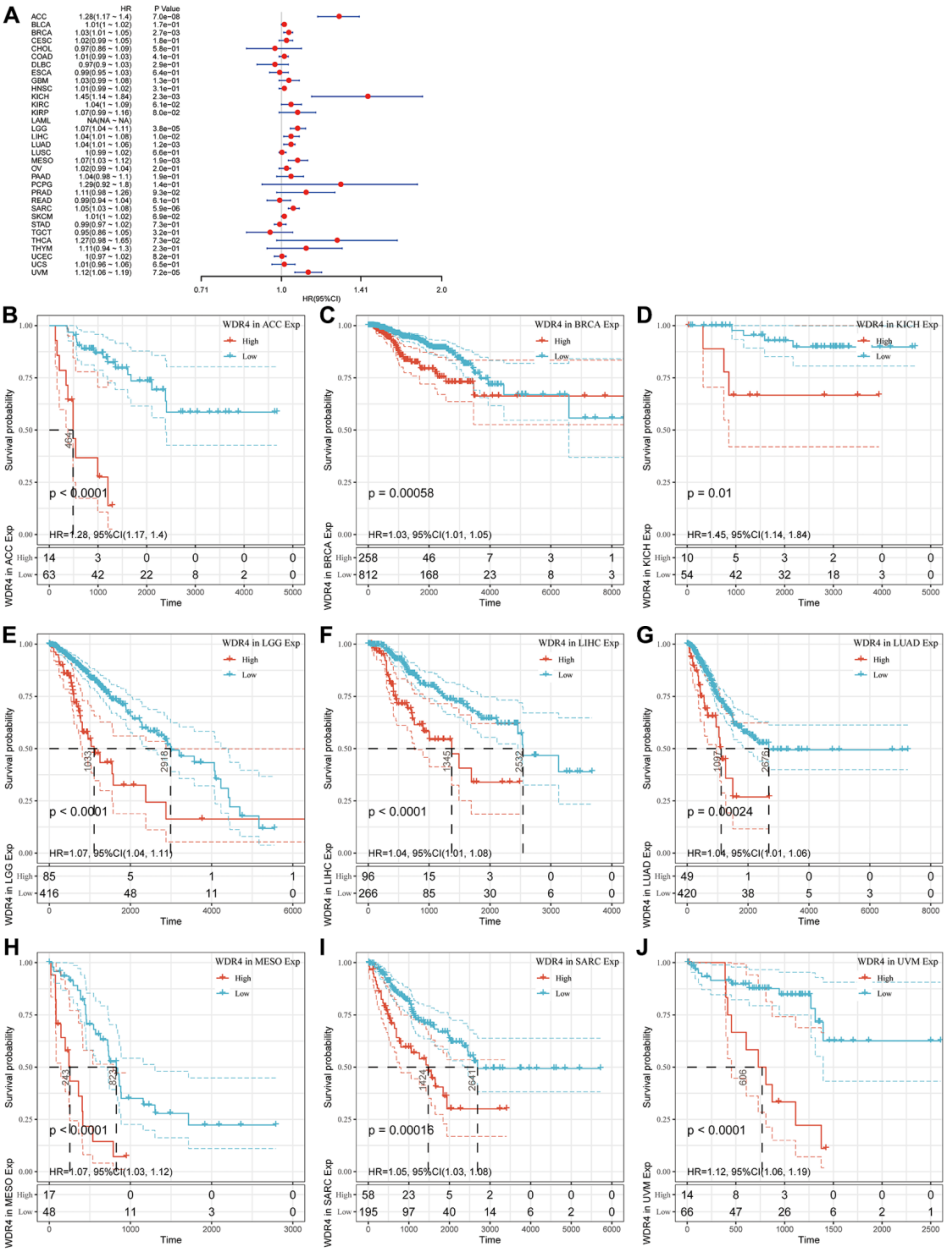
**Association of WDR4 expression with patient disease-specific survival (DSS).** (**A**) The forest plot shows the relationship of WDR4 expression with DSS. (**B**–**J**) Kaplan-Meier analyses show the association between WDR4 expression and DSS.

The Cox regression analysis of DSS indicated that high WDR4 expression is a risk factor in ACC (*P* < .001), BLCA (*P* < .001), BRCA (*P* < .001), KICH (*P* < .001), KIRC (*P* < .001), LGG (*P* < .001), LIHC (*P* < .001), LUAD (*P* < .001), MESO (*P* < .001), SARC (*P* < .001), SKCM (*P* < .001) and UVM (*P* < .001), as illustrated in Figure 4A. KM analysis revealed that patients with high WDR4 expression had worse DSS than those with low WDR4 expression in ACC (*P* < .001), BRCA (*P* < .001), KICH (*P* = .01), LGG (*P* < .001), LIHC (*P* < .001), LUAD (*P* < .001), MESO (*P* < .001), SARC (*P* < .001) and UVM (*P* < .001), as illustrated in Figure 4B–4J.

**Figure 5:**
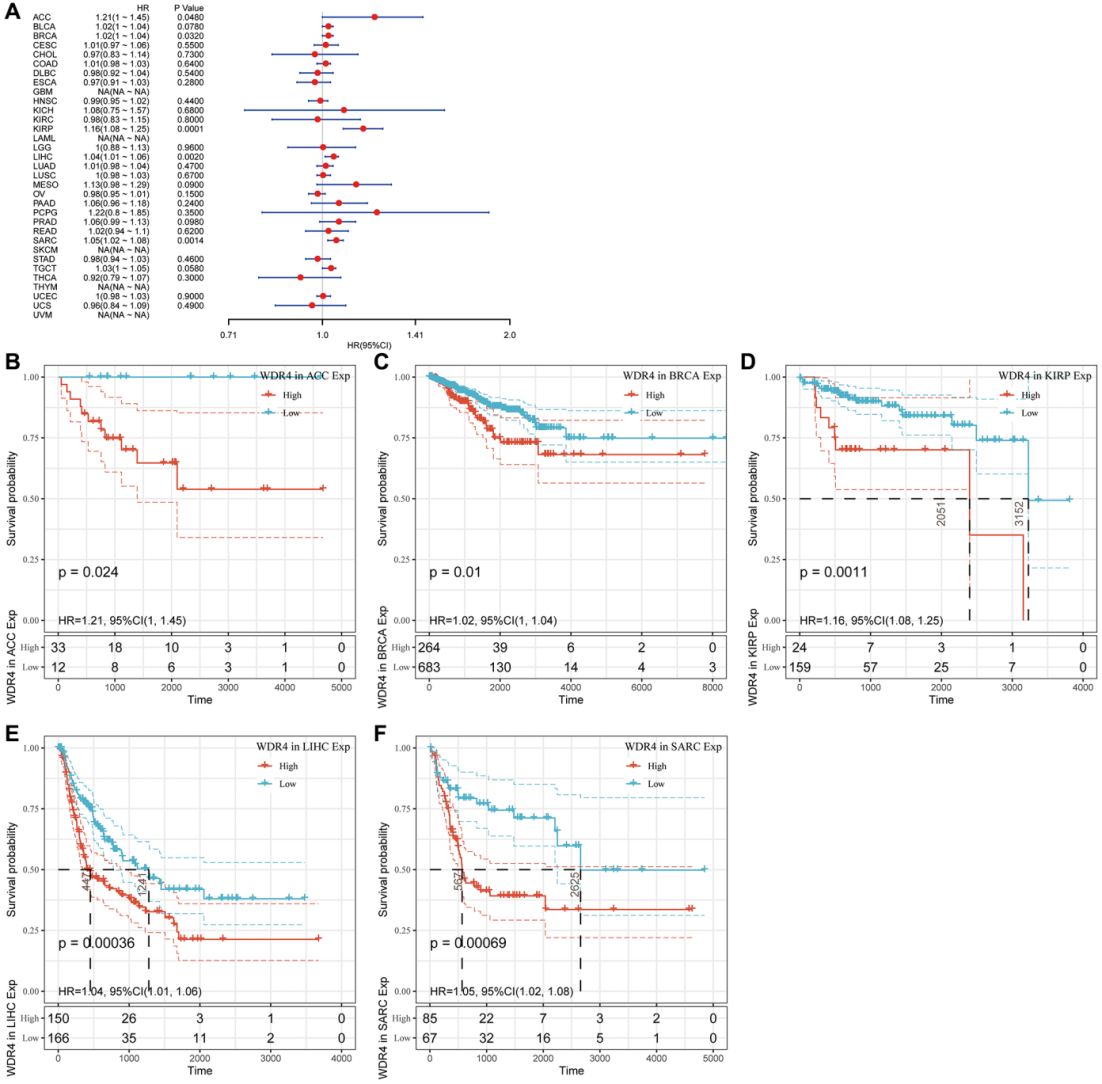
**Association of WDR4 expression with patient disease-free interval (DFI).** (**A**) The forest plot shows the relationship of WDR4 expression with DFI. (**B**–**F**) Kaplan-Meier analyses show the association between WDR4 expression and DFI.

The Cox regression analysis of DFI indicated that higher WDR4 expression was a risk factor in ACC (*P* = .048), BRCA (*P* = .032), KIRP (*P* < .001), LIHC (*P* = .002), and SARC (*P* = .0014), as illustrated in Figure 5A. KM analysis showed that patients with higher WDR4 expression had a poorer DFI than those with lower WDR4 expression in ACC (*P* = .024), BRCA (*P* = .01), KIRP (*P* = .0011), LGG (*P* < .001), LIHC (*P* < .001) and SARC (*P* < .001), as illustrated in Figure 5B–5F.

**Figure 6:**
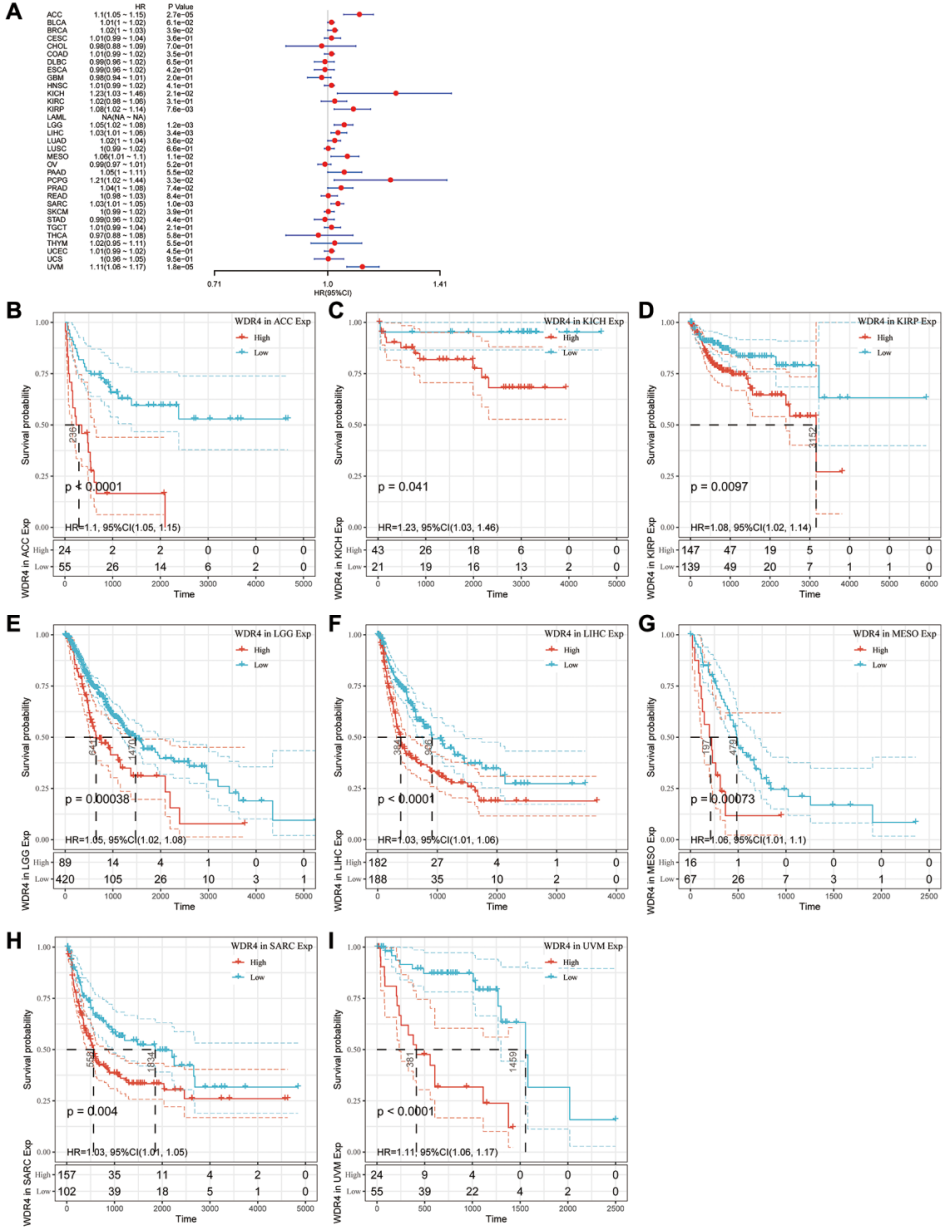
**Association of WDR4 expression with patient progression-free interval (PFI).** (**A**) The forest plot shows the relationship of WDR4 expression with PFI. (**B**–**I**) Kaplan-Meier analyses show the association between WDR4 expression and PFI.

The Cox regression analysis of PFI revealed that higher WDR4 expression is a risk factor in ACC (*P* < .001), BLCA (*P* < .001), BRCA (*P* < .001), KIHC (*P* < .001), KIRP (*P* < .001), LGG (*P* < .001), LIHC (*P* < .001), LUAD (*P* < .001), MESO (*P* < .001), PAAD (*P* < .001), PCPG (*P* < .001), PRAD (*P* < .001), SARC (*P* < .001) and UVM (*P* < .001), as illustrated in Figure 6A. KM analysis indicated that patients with high WDR4 expression had a worse PFI than those with lower WDR4 expression in ACC (*P* < .001), KICH (*P* < .001), KIRP (*P* = .0011), LGG (*P* < .001), LIHC (*P* < .001), MESO (*P* = .004) and SARC (*P* < .001), as illustrated in Figure 6B–6I.

### The WDR4 level was linked to the level of immune infiltration and immune markers

**Figure 7:**
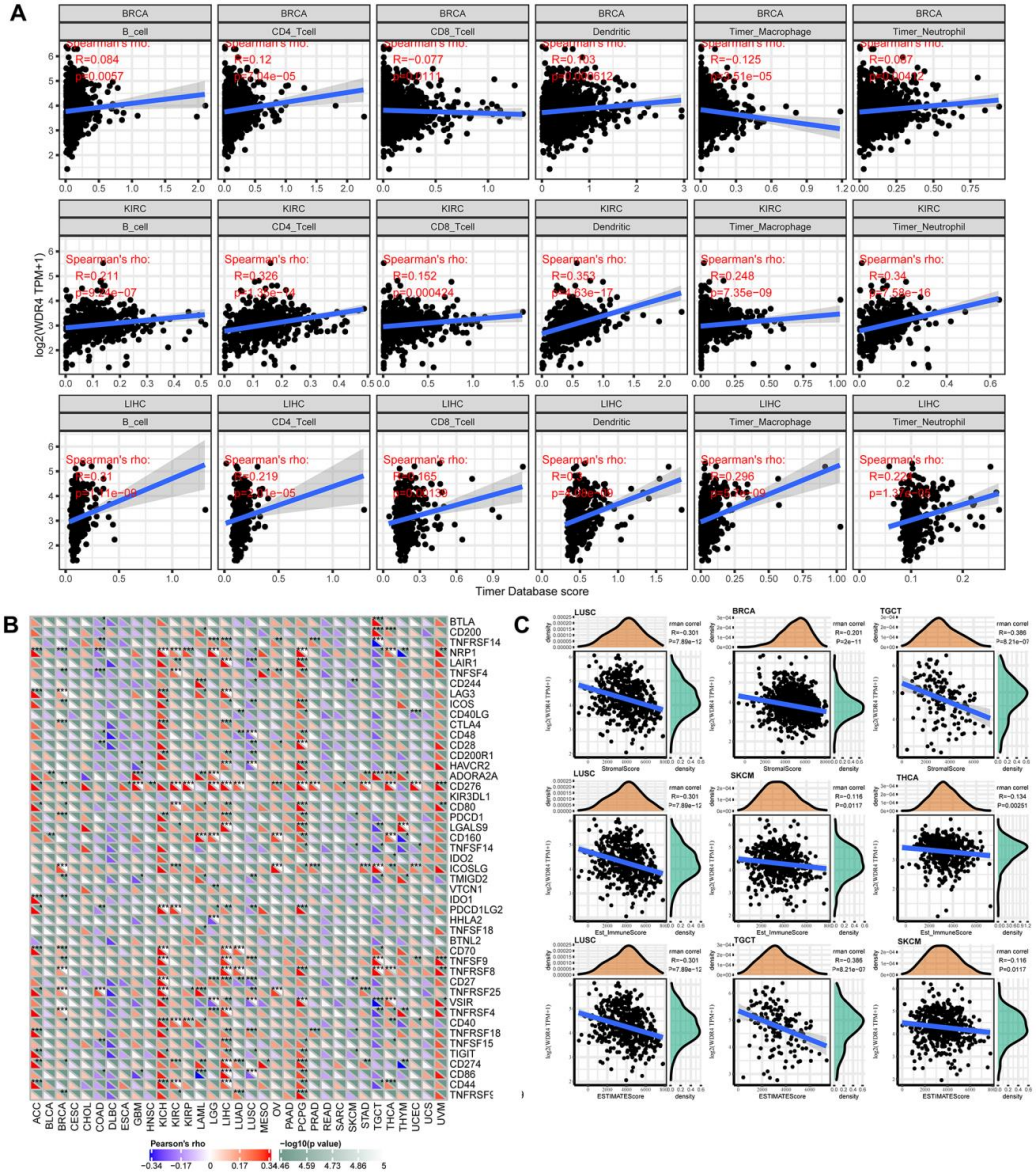
**WDR4 expression is correlated with cancer immunity.** (**A**) TIMER predicts that the WDR4 level is related to the degree of immune infiltration within BRCA, CHOL and HNSC. (**B**) The heat map represents the relationship between 47 immune checkpoint genes and the gene expression of WDR4. For each pair, the right triangle is coloured to represent the *P*-value; the bottom left o is coloured to indicate the Spearman correlation coefficient. ^*^P < .05; ^**^P < .01; ^***^P < .001. (**C**) Relationship between gene expression and the StromalScore, Est_ImmuneScore and ESTIMATEScore.

Tumour-infiltrating lymphocytes (TILs) can be independent predictors of SLN (sentinel lymph node) involvement and cancer survival outcomes. We observed an association between WRD4 expression levels and the levels of immune infiltration in numerous carcinoma types using TIMER analysis. It was discovered that the WDR4 levels were significantly correlated with the infiltration levels of CD4+ T and CD8+ T cells in 21 types of carcinoma, B cells in 14 types, neutrophils in 15 types, macrophages in 14 types, and dendritic cells in 19 types. As WDR4 expression had prognostic value in BRCA, KIRC, and LIHC, the correlation between WDR4 expression levels and the degree of immune infiltration in BRCA, KIRC, and LIHC is shown in Figure 7A.

To investigate the relationship between WDR4 expression and various infiltrating immune cells, the associations between WDR4 expression and immune markers in a range of immunocytes were studied, as shown in Figure 7B. We discovered that WDR4 expression was associated with the expression levels of CD276 in ACC, BRCA, GBM, ESCA, HNSC, LGG, KIRP, KIRC, LIHC, OV, PCPG, PRAD, LUAD, SARC, THCA, STAD, UVM, and UCEC, signifying that WDR4 may affect the immune response in these tumours.

### Correlation analysis with immune score

WDR4 expression was typically correlated with the stromal score in LUCS, BRCA, and TGCT, the Est_ImmuneScore in LUCS, SKCM, and THCA, and the ESTIMATE score in LUSC, TGCT, and SKCM (Figure 7C).

**Figure 8:**
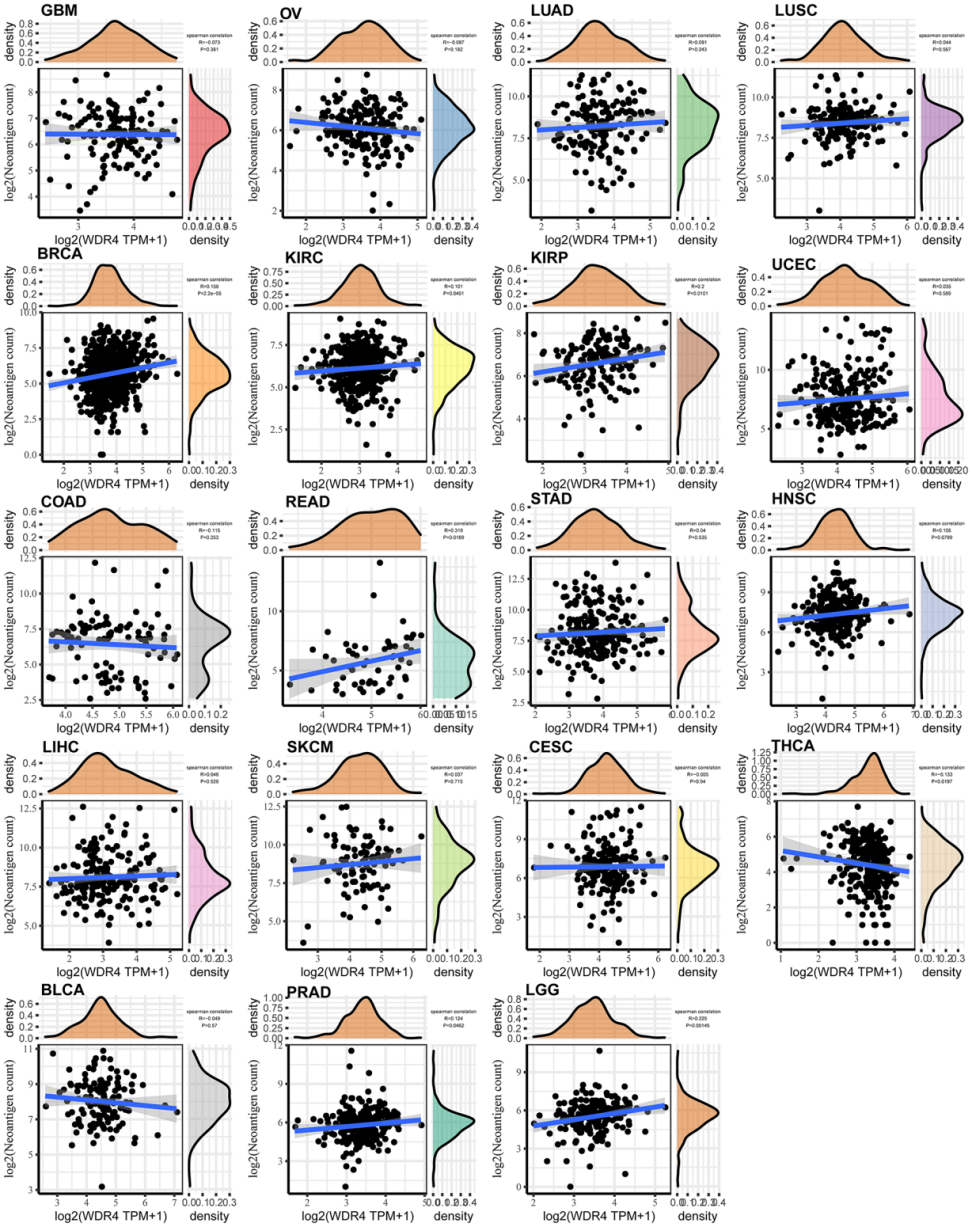
Relationship between the number of neoantigens and gene expression in each tumour.

As shown in Figure 8, WDR4 expression was negatively associated with neoantigens in GBM, OV, COAD, CESC, THCA, and BLCA but positively correlated with neoantigens in LUAD, LUSC, BRCA, KIRC, KIRP, UCEC, READ, STAD, HNSC, LIHC, SKCM, PRAD, and LGG.

**Figure 9:**
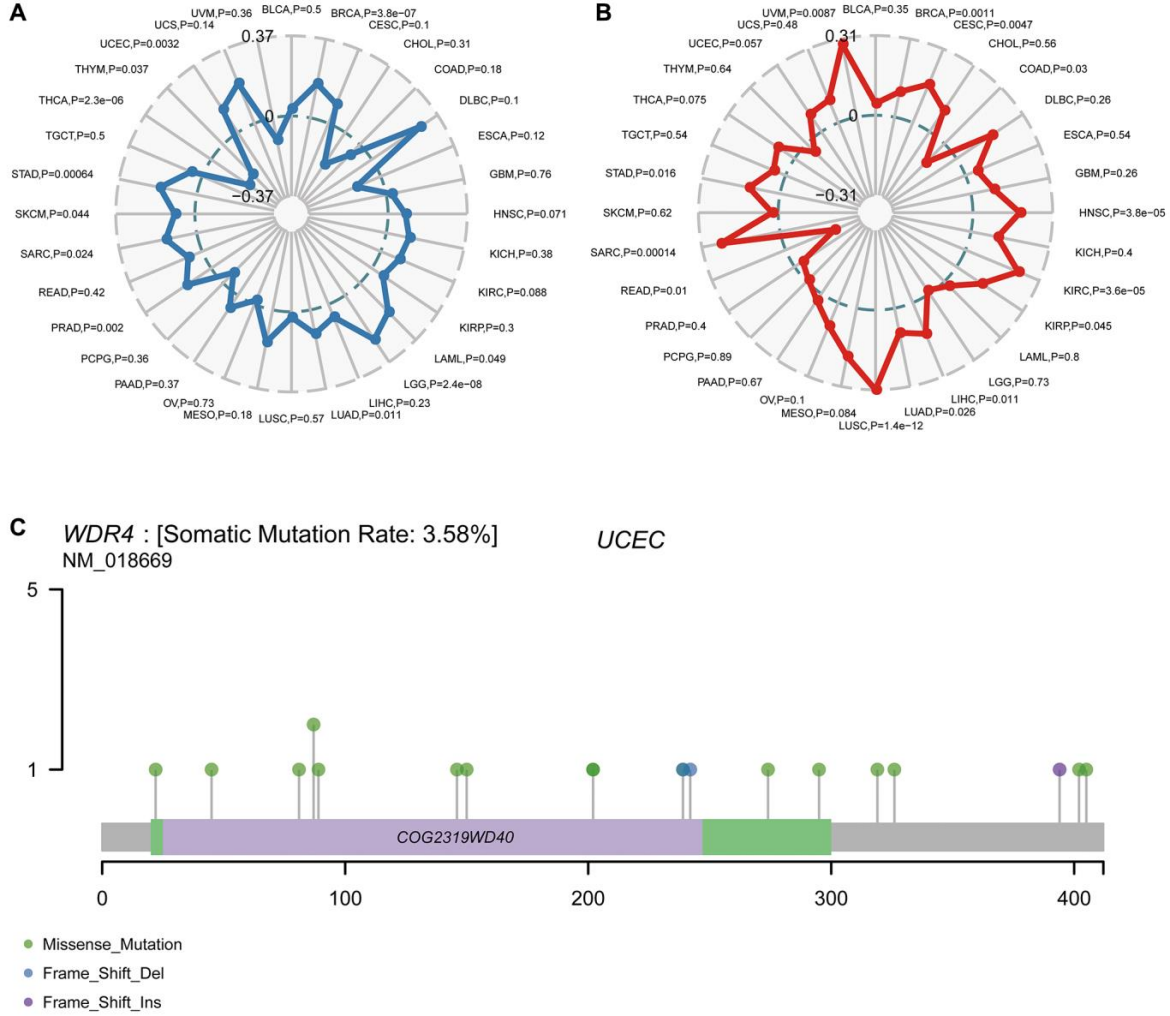
**Correlation of WDR4 expression with TMB and MSI and mutation pattern of the WDR4 gene in tumour samples.** (**A**) The radar chart displays the overlap between WDR4 and TMB. The number represents the Spearman correlation coefficient. (**B**) The radar chart displays the overlap between WDR4 and MSI. The number represents the Spearman correlation coefficient. (**C**) Mutation of WDR4 in UCSC.

We found that WDR4 expression was positively associated with TMB in UCEC (*P* = .0032), STAD (*P* < .001), SKCM (*P* = .044), SARC (*P* = .024), PRAD (*P* = .002), LUAD (*P* = .011), LGG (*P* < .001), LAML (*P* = .049) and BRCA (*P* < .001) but negatively associated with THYM (*P* = .037) and THCA (*P* < .001), as illustrated in Figure 9A. We further discovered that the WDR4 level was positively associated with MSI in UVM (*P* = .0087), STAD (*P* = .016), SARC (*P* < .001), LUSC (*P* < .001), LUAD (*P* = .026), LIHC (*P* = .011), KIRP (*P* = .045), KIRC (*P* < .001), HNSC (*P* < .001), CESC (*P* < .001) and BRCA (*P* = .0011) but negatively associated with READ (*P* = .01) and COAD (*P* = .03), as illustrated in Figure 9B.

**Figure 10:**
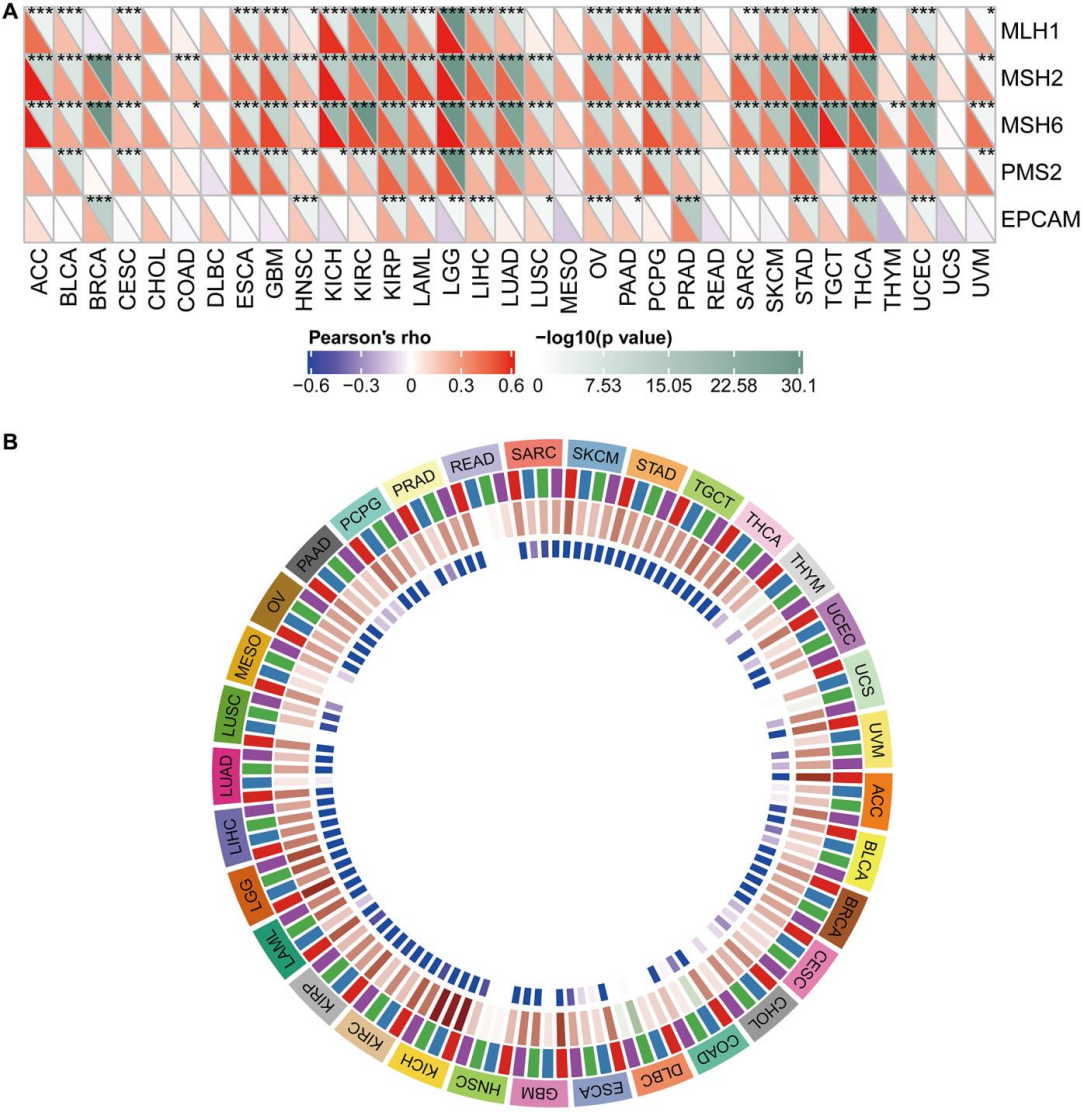
**Relationship between WDR4 expression and MMRS and methyltransferase in various tumour samples.** (**A**) Relationship between WDR4 expression and mutation of 5 MMR genes. (**B**) Relationship between 4 methyltransferases and WDR4 expression.

Figure 9C shows the typical presentation of UCEC, in which the somatic mutation rate of WDR4 is 3.58%. As shown in Figure 10A, the expression of WDR4 was significantly correlated with mutations in 5 MMR genes (MLH1, MLH2, MLH6, PMS2, EPCAM) in several cancer types. In addition, a close relationship was observed between WDR4 expression and mutations in 4 methyltransferases in several cancer types (Figure 10B).

### Functional analysis

**Figure 11:**
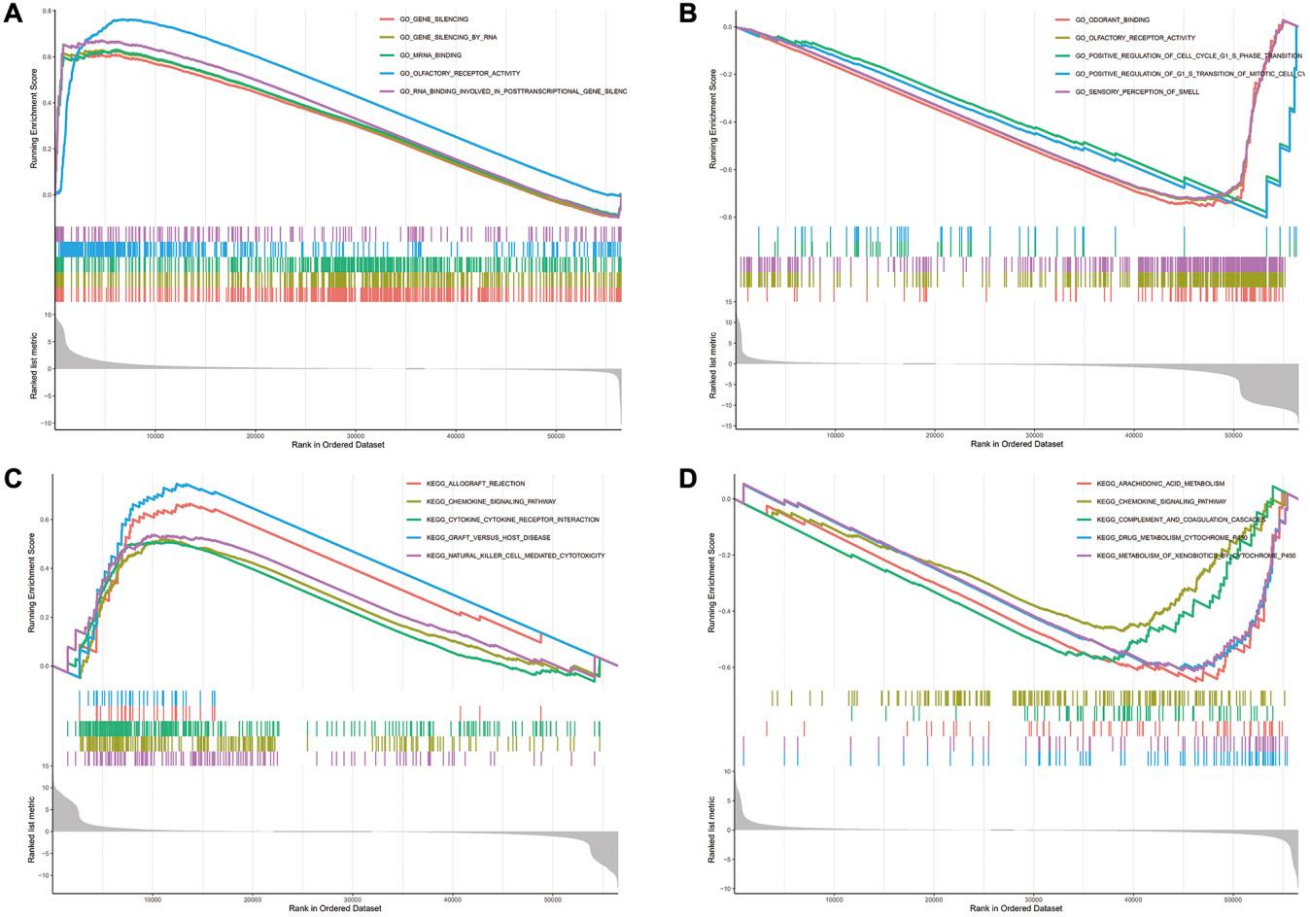
**The enrichment results of GO and KEGG pathways in the high expression group and the low expression group.** (**A**–**D**) The enrichment results of GO and KEGG pathways in the high expression group and the low expression group.

The biological consequence of WDR4 expression was evaluated by means of GSEA. In SKCM, WDR4 levels were associated with enrichment of the following GO terms:

GO_EPIDERMIS_DEVELOPMENT, GO_LEUKOCYTE_MIGRATION, GO_NEGATIVE_REGULATION_OF_IMMUNE_SYSTEM_PROCESS, GO_POSITIVE_REGULATION_OF_CELL_ADHESION GO_POSITIVE_REGULATION_OF_CYTOKIN_PRODUCTION. The following KEGG terms also were also significantly associated with WDR4 levels: KEGG_ARACHIDONIC_ACID_METABOLISM, KEGG_CHEMOKINE_SIGNALING_PATHWAY, KEGG_COMPLEMENT_AND_COAGULATION_CASCADES, KEGG_DRUG_METABOLISM_CYTOCHROME_P450 and KEGG_METABOLISM_OF_XENOBIOTICS_BY_CTTDCHROME_P450. In UVM, WDR4 was associated with enrichment of the following GO terms:

GO_CILIUM_MOVEMENT, GO_NON_MOTILE_CILIUM, GO_REGULATION_OF_CELL_MIGRATION_INVOLVED_IN_SPROUTING_ANGIOGI, GO_REGULATION_OF_VASCULAR_SMOOTH_MUSCLE_CELL_PROLIFERATION and GO_RNA_3_END_PROCESSING. The following KEGG terms also presented significant enrichment: KEGG_ALLOGRAFT_REJECTION, KEGG_CHEMOKINE_SIGNALING_PATHWAY, KEGG_CYTOKINE_CYTOKINE_RECEPTOR_INTERACTION, KEGG_DRAFT_VERSUS_HOST_DISEASE, and KEGG_NATURAL_KILLER_CELL_MEDIATED_CYTOTOXICITY. These pathways are shown in Figure 11.

## DISCUSSION

With the development of knowledge and medical expertise, clinicians can improve the cure rate of tumour patients by surgical operation, chemotherapy, endocrine therapy, or targeted therapy [23, 24]. Nevertheless, tumour management is not always adequate. It is essential to identify tumour-specific targets or characteristics for individualized treatment to increase the chance of curing cancer patients [25]. We aimed to conduct a comprehensive pan-cancer analysis to highlight the vital role of WDR4 in various types of malignancy. We obtained a large dataset of different types of cancer from the GTEX and TCGA databases to analyze the abnormal expression of WDR4 in different types of cancer. By CCLE, a detailed investigation of the gene expression levels in the various cancer types was conducted, and the results might inspire future cell experiments. We discovered that the expression of WDR4 differed among various cancers, and abnormal expression of WDR4 was revealed to be a prognostic factor in certain types of malignancy by both Cox and KM survival analyses. In our research, higher WDR4 levels were associated with a worse prognosis in ACC, KIRC, LGG, LIHC, LUAD, MESO, READ, SARC, and UVM patients.

Tumour-infiltrating lymphocytes (TILs) are independent predictors of SLN involvement and cancer patient survival outcomes [26, 27]. Furthermore, immunotherapy has improved the efficacy of tumour treatment [28–30]. We found by TIMER analysis that WDR4 levels are significantly associated with the infiltration of CD8+ and CD4+ T cells in in 21 types of cancer, B cells in 14 types, dendritic cells in 19 types, neutrophils in 15 types, and macrophages in 14 types. Moreover, the expression of WDR4 was corelated with both the number of infiltrating immune cells and the patient prognosis in BRCA, KIRC, and LIHC. B7-H3 (CD276) is a vital immune checkpoint member of the B7 and CD28 families. B7-H3 is highly overexpressed in a wide variety of human solid malignances and is frequently related to both poor patient prognosis and poor clinical results [31, 32]. Amori et al. discovered that high B7-H3 expression was more often detected in patients with metastatic prostate carcinoma than in individuals with localized carcinoma; thus, B7-H3 might be a beneficial biomarker for extremely aggressive metastatic prostate cancer [33]. In our study, we discovered that WDR4 expression levels were correlated with the expression levels of CD276 in ESCA, ACC, BRCA, GBM, OV, KIRC, HNSC, KIRP, LIHC, LGG, LUAD, PCPG, STAD, PRAD, SARC, THCA, UVM, and UCEC, suggesting that WDR4 may regulate the immune response in these tumour types.

Genetic alterations are the main cause of malignancy. Specific gene mutations can predictors of patient prognosis and of the patient’s response to treatment. The adaptive immune system recognizes and identifies cancers due to nonself-neoantigens associated with somatic mutations. The TMB level influences the production of immunogenic peptides, therefore influencing the response to immune checkpoint inhibitors [34–36]. In this study, we discovered that WDR4 expression was equally associated with TMB and MSI in STAD, SARC PRAD, LUAD, and BRCA. Nevertheless, additional studies are needed to determine whether WDR4 can be used as a predictive biomarker for immunotherapy response in patients with these cancers. Hence, the results of the current study provide a basis for further investigating the connection between WDR4 expression levels and cancer immunity.

Our thorough pan-cancer investigation illustrated the role of WDR4 in tumour cells and tissues. Furthermore, we discovered that WDR4 levels can serve as a valuable prognostic biomarker for some types of tumour. According to the findings presented in the current report, the WDR4 level is associated with cancer immunity. Similarly, our latest integrated omics-based workflow could be adopted to identify novel targets for carcinoma treatment.

## References

[1] Bray F, Ferlay J, Soerjomataram I, Siegel RL, Torre LA, Jemal A. Global cancer statistics 2018: GLOBOCAN estimates of incidence and mortality worldwide for 36 cancers in 185 countries. CA Cancer J Clin. 2018; 68:394–424. 10.3322/caac.21492 30207593

[2] Schwaederle M, Zhao M, Lee JJ, Lazar V, Leyland-Jones B, Schilsky RL, Mendelsohn J, Kurzrock R. Association of Biomarker-Based Treatment Strategies With Response Rates and Progression-Free Survival in Refractory Malignant Neoplasms: A Meta-analysis. JAMA Oncol. 2016; 2:1452–59. 10.1001/jamaoncol.2016.2129 27273579

[3] Weinstein JN, Collisson EA, Mills GB, Shaw KR, Ozenberger BA, Ellrott K, Shmulevich I, Sander C, Stuart JM, and Cancer Genome Atlas Research Network. The Cancer Genome Atlas Pan-Cancer analysis project. Nat Genet. 2013; 45:1113–20. 10.1038/ng.2764 24071849 PMC3919969

[4] Gentles AJ, Newman AM, Liu CL, Bratman SV, Feng W, Kim D, Nair VS, Xu Y, Khuong A, Hoang CD, Diehn M, West RB, Plevritis SK, Alizadeh AA. The prognostic landscape of genes and infiltrating immune cells across human cancers. Nat Med. 2015; 21:938–45. 10.1038/nm.3909 26193342 PMC4852857

[5] Wang Y, Wang X, Xiong Y, Li CD, Xu Q, Shen L, Chandra Kaushik A, Wei DQ. An Integrated Pan-Cancer Analysis and Structure-Based Virtual Screening of GPR15. Int J Mol Sci. 2019; 20:6226. 10.3390/ijms20246226 31835584 PMC6940937

[6] Huang ZL, Li W, Chen QF, Wu PH, Shen LJ. Eight key long non-coding RNAs predict hepatitis virus positive hepatocellular carcinoma as prognostic targets. World J Gastrointest Oncol. 2019; 11:983–97. 10.4251/wjgo.v11.i11.983 31798779 PMC6883184

[7] Armendáriz-Castillo I, López-Cortés A, García-Cárdenas J, Guevara-Ramírez P, Leone PE, Pérez-Villa A, Yumiceba V, Zambrano AK, Guerrero S, Paz-Y-Miño C. TCGA Pan-Cancer Genomic Analysis of Alternative Lengthening of Telomeres (ALT) Related Genes. Genes (Basel). 2020; 11:834. 10.3390/genes11070834 32708340 PMC7397314

[8] Machnik M, Cylwa R, Kiełczewski K, Biecek P, Liloglou T, Mackiewicz A, Oleksiewicz U. The expression signature of cancer-associated KRAB-ZNF factors identified in TCGA pan-cancer transcriptomic data. Mol Oncol. 2019; 13:701–24. 10.1002/1878-0261.12407 30444046 PMC6442004

[9] Xu WX, Zhang J, Hua YT, Yang SJ, Wang DD, Tang JH. An Integrative Pan-Cancer Analysis Revealing LCN2 as an Oncogenic Immune Protein in Tumor Microenvironment. Front Oncol. 2020; 10:605097. 10.3389/fonc.2020.605097 33425761 PMC7786136

[10] Zhao Y, Zhang M, Pu H, Guo S, Zhang S, Wang Y. Prognostic Implications of Pan-Cancer CMTM6 Expression and Its Relationship with the Immune Microenvironment. Front Oncol. 2021; 10:585961. 10.3389/fonc.2020.585961 33552963 PMC7855963

[11] Ma WF, Boudreau HE, Leto TL. Pan-Cancer Analysis Shows *TP53* Mutations Modulate the Association of NOX4 with Genetic Programs of Cancer Progression and Clinical Outcome. Antioxidants (Basel). 2021; 10:235. 10.3390/antiox10020235 33557266 PMC7915715

[12] Ghoshdastider U, Rohatgi N, Mojtabavi Naeini M, Baruah P, Revkov E, Guo YA, Rizzetto S, Wong AML, Solai S, Nguyen TT, Yeong JPS, Iqbal J, Tan PH, et al. Pan-Cancer Analysis of Ligand-Receptor Cross-talk in the Tumor Microenvironment. Cancer Res. 2021; 81:1802–12. 10.1158/0008-5472.CAN-20-2352 33547160

[13] Cheng S, Li Z, Gao R, Xing B, Gao Y, Yang Y, Qin S, Zhang L, Ouyang H, Du P, Jiang L, Zhang B, Yang Y, et al. A pan-cancer single-cell transcriptional atlas of tumor infiltrating myeloid cells. Cell. 2021; 184:792–809.e23. 10.1016/j.cell.2021.01.010 33545035

[14] Luo Z, Liu W, Sun P, Wang F, Feng X. Pan-cancer analyses reveal regulation and clinical outcome association of the shelterin complex in cancer. Brief Bioinform. 2021. [Epub ahead of print]. 10.1093/bib/bbaa441 33497432

[15] Lin S, Liu Q, Lelyveld VS, Choe J, Szostak JW, Gregory RI. Mettl1/Wdr4-Mediated m^7^G tRNA Methylome Is Required for Normal mRNA Translation and Embryonic Stem Cell Self-Renewal and Differentiation. Mol Cell. 2018; 71:244–55.e5. 10.1016/j.molcel.2018.06.001 29983320 PMC6086580

[16] Rastegari E, Kajal K, Tan BS, Huang F, Chen RH, Hsieh TS, Hsu HJ. WD40 protein Wuho controls germline homeostasis via TRIM-NHL tumor suppressor Mei-p26 in *Drosophila*. Development. 2020; 147:dev182063. 10.1242/dev.182063 31941704 PMC7375833

[17] Michaud J, Kudoh J, Berry A, Bonne-Tamir B, Lalioti MD, Rossier C, Shibuya K, Kawasaki K, Asakawa S, Minoshima S, Shimizu N, Antonarakis SE, Scott HS. Isolation and characterization of a human chromosome 21q22.3 gene (WDR4) and its mouse homologue that code for a WD-repeat protein. Genomics. 2000; 68:71–79. 10.1006/geno.2000.6258 10950928

[18] Wang YT, Chen J, Chang CW, Jen J, Huang TY, Chen CM, Shen R, Liang SY, Cheng IC, Yang SC, Lai WW, Cheng KH, Hsieh TS, et al. Ubiquitination of tumor suppressor PML regulates prometastatic and immunosuppressive tumor microenvironment. J Clin Invest. 2017; 127:2982–97. 10.1172/JCI89957 28691927 PMC5531412

[19] Lee CC, Hsieh TS. Wuho/WDR4 deficiency inhibits cell proliferation and induces apoptosis via DNA damage in mouse embryonic fibroblasts. Cell Signal. 2018; 47:16–26. 10.1016/j.cellsig.2018.03.007 29574139

[20] Li T, Fan J, Wang B, Traugh N, Chen Q, Liu JS, Li B, Liu XS. TIMER: A Web Server for Comprehensive Analysis of Tumor-Infiltrating Immune Cells. Cancer Res. 2017; 77:e108–10. 10.1158/0008-5472.CAN-17-0307 29092952 PMC6042652

[21] Li B, Severson E, Pignon JC, Zhao H, Li T, Novak J, Jiang P, Shen H, Aster JC, Rodig S, Signoretti S, Liu JS, Liu XS. Comprehensive analyses of tumor immunity: implications for cancer immunotherapy. Genome Biol. 2016; 17:174. 10.1186/s13059-016-1028-7 27549193 PMC4993001

[22] Killock D. TMB - a histology-agnostic predictor of the efficacy of ICIs? Nat Rev Clin Oncol. 2020; 17:718. 10.1038/s41571-020-00438-0 32968242

[23] Pelkonen M, Luostari K, Tengström M, Ahonen H, Berdel B, Kataja V, Soini Y, Kosma VM, Mannermaa A. Low expression levels of hepsin and TMPRSS3 are associated with poor breast cancer survival. BMC Cancer. 2015; 15:431. 10.1186/s12885-015-1440-5 26014348 PMC4445813

[24] Luo SP, Zhang J, Wu QS, Lin YX, Song CG. Association of Axillary Lymph Node Evaluation With Survival in Women Aged 70 Years or Older With Breast Cancer. Front Oncol. 2021; 10:596545. 10.3389/fonc.2020.596545 33585213 PMC7877252

[25] Andre F, Mardis E, Salm M, Soria JC, Siu LL, Swanton C. Prioritizing targets for precision cancer medicine. Ann Oncol. 2014; 25:2295–303. 10.1093/annonc/mdu478 25344359

[26] Khammari A, Knol AC, Nguyen JM, Bossard C, Denis MG, Pandolfino MC, Quéreux G, Bercegeay S, Dréno B. Adoptive TIL transfer in the adjuvant setting for melanoma: long-term patient survival. J Immunol Res. 2014; 2014:186212. 10.1155/2014/186212 24741578 PMC3987883

[27] Kitsou M, Ayiomamitis GD, Zaravinos A. High expression of immune checkpoints is associated with the TIL load, mutation rate and patient survival in colorectal cancer. Int J Oncol. 2020; 57:237–48. 10.3892/ijo.2020.5062 32468013 PMC7252459

[28] Hindson J. PD1 blockade for advanced MSI-H CRC. Nat Rev Gastroenterol Hepatol. 2021; 18:82. 10.1038/s41575-021-00415-7 33437018

[29] Lin G, Zhang Y, Yu L, Wu D. Cytotoxic effect of CLL-1 CAR-T cell immunotherapy with PD-1 silencing on relapsed/refractory acute myeloid leukemia. Mol Med Rep. 2021; 23:208. 10.3892/mmr.2021.11847 33495835 PMC7830996

[30] Sethi M, Garg V, Lee JB, Yang S. PD1 inhibitor induced inverse lichenoid eruption: a case series. Dermatol Online J. 2020; 26:13030/qt66b8298z. 33423421

[31] Picarda E, Ohaegbulam KC, Zang X. Molecular Pathways: Targeting B7-H3 (CD276) for Human Cancer Immunotherapy. Clin Cancer Res. 2016; 22:3425–31. 10.1158/1078-0432.CCR-15-2428 27208063 PMC4947428

[32] Inamura K, Takazawa Y, Inoue Y, Yokouchi Y, Kobayashi M, Saiura A, Shibutani T, Ishikawa Y. Tumor B7-H3 (CD276) Expression and Survival in Pancreatic Cancer. J Clin Med. 2018; 7:172. 10.3390/jcm7070172 29996538 PMC6069252

[33] Inamura K, Yokouchi Y, Kobayashi M, Ninomiya H, Sakakibara R, Nishio M, Okumura S, Ishikawa Y. Relationship of tumor PD-L1 (CD274) expression with lower mortality in lung high-grade neuroendocrine tumor. Cancer Med. 2017; 6:2347–56. 10.1002/cam4.1172 28925087 PMC5633594

[34] Martincorena I, Campbell PJ. Somatic mutation in cancer and normal cells. Science. 2015; 349:1483–89. 10.1126/science.aab4082 26404825

[35] Sanz-Garcia E, Argiles G, Elez E, Tabernero J. BRAF mutant colorectal cancer: prognosis, treatment, and new perspectives. Ann Oncol. 2017; 28:2648–57. 10.1093/annonc/mdx401 29045527

[36] Havel JJ, Chowell D, Chan TA. The evolving landscape of biomarkers for checkpoint inhibitor immunotherapy. Nat Rev Cancer. 2019; 19:133–50. 10.1038/s41568-019-0116-x 30755690 PMC6705396

